# Molecular basis of urostyle development in frogs: genes and gene regulation underlying an evolutionary novelty

**DOI:** 10.1098/rsob.240111

**Published:** 2024-08-28

**Authors:** Gayani Senevirathne, Neil H. Shubin

**Affiliations:** ^1^ Human Evolutionary Biology, Harvard University, Cambridge, MA 02138, USA; ^2^ Organismal Biology and Anatomy, University of Chicago Biological Sciences Division, Chicago, IL 60637, USA

**Keywords:** hypochord, RNA-seq, ATAC-seq, T-box genes, metamorphosis

## Abstract

Evolutionary novelties entail the origin of morphologies that enable new functions. These features can arise through changes to gene function and regulation. One key novelty is the fused rod at the end of the vertebral column in anurans, the urostyle. This feature is composed of a coccyx and a hypochord, both of which ossify during metamorphosis. To elucidate the genetic basis of these features, we used laser capture microdissection of these tissues and did RNA-seq and ATAC-seq at three developmental stages in tadpoles of *Xenopus tropicalis*. RNA-seq reveals that the coccyx and hypochord have two different molecular signatures. Neuronal (*TUBB3*) and muscle markers (*MYH3*) are upregulated in coccygeal tissues, whereas T-box genes (*TBXT*, *TBXT.2*), corticosteroid stress hormones (*CRCH.1*) and matrix metallopeptidases (*MMP1*, *MMP8* and *MMP13*) are upregulated in the hypochord. ATAC-seq reveals potential regulatory regions that are observed in proximity to candidate genes that regulate ossification identified from RNA-seq. Even though an ossifying hypochord is only present in anurans, this ossification between the vertebral column and the notochord resembles a congenital vertebral anomaly seen prenatally in humans caused by an ectopic expression of the *TBXT*/*TBXT.2* gene. This work opens the way to functional studies that can elucidate anuran *bauplan* evolution.

## 1. Introduction

Phenotypic and genotypic changes from an ancestral condition undergird the evolution of ‘key innovations’ [1]. Phenotypic changes of a novel structure reflect changes in the corresponding genotypic/gene regulatory networks [2–5]. The anuran (frog and toad) urostyle, composed of a coccyx and a hypochord, is morphologically unique from the rest of the vertebrates because of the contribution of an ossifying hypochord, and is therefore considered a structural novelty [6–10]. The coccyx is derived from the paraxial mesoderm, gives rise to the caudal vertebrae [7,11],[12] which subsequently undergo endochondral ossification and fuse together during metamorphosis [6]. The amphibian hypochord, thought to be derived from either endoderm [13–15] or superficial mesoderm [16], is a thin embryonic rod, which degenerates in the rest of anamniotes during early embryonic development but is retained only in frogs and undergoes endochondral ossification during the metamorphic climax [6–10].

Embryonic hypochord in anamniotes is known to have a function in remodelling the dorsal aorta. Previous work [6] has shown how the ossifying hypochord may also play a role in modifying the dorsal aorta by occluding it at the posterior-most end of the hypochord and remodelling it to form two branches, which enter the forelimbs and hind limbs, respectively. Senevirathne *et al.* [6] speculated that the ossifying hypochord has a role in the evolution of the unique anuran *bauplan*, which has been conserved in anurans for more than 200 million years [2,17].

The morphological changes of the urostyle are well studied [6,8,9]; however, the molecular mechanisms underlying this unique structure have remained obscure to date. Here, we investigate transcriptomic and gene regulatory networks in the developing urostyle by combining RNA-seq and ATAC-seq approaches. Using previously published morphology work [6] as a framework to identify targeted cells, we used laser capture microdissection (LCM) to reveal the transcriptomics and epigenomics of the two tissue types: coccyx and hypochord.

Paraxial mesoderm-derived coccygeal cells are undifferentiated mesenchymal cells; they undergo chondrification and ossification prior to the initiation of the metamorphic climax [6,7] and could be following a similar gene regulatory network as connective tissues and bones in vertebrates. However, the ossifying hypochord initiates ossification at the onset of metamorphosis. The origin of amphibian hypochordal cells has been hypothesized to be from the endoderm [13–15] or the superficial mesoderm [16]. Regardless of which germ layer it is derived from, hypochord also undergoes endochondral ossification only in anurans, and the hypochord and the coccyx appear to have a conserved regulatory network. Here, we compare the gene expression patterns of coccygeal and hypochordal cells to identify similar/different pathways between the two tissue types, which are derived from two different cell populations.

Using *Xenopus tropicalis* as our model organism, we address the following questions through this work. Why does the hypochord only ossify in anurans? What are the similarities/differences between the hypochordal and coccygeal molecular pathways? Which genes switch on/off during metamorphosis? By identifying the underlying changes in the genes and gene regulatory networks, our work begins to shed light on the potential genotypic changes underlying a structural novelty.

## 2. Material and methods

Different stages of *X. tropicalis* tadpoles were purchased from the National Xenopus Resource (NXR) at the Marine Biological Laboratory (MBL), Woods Hole, MA. Comparisons were made across three significant life-history stages to analyse genes and gene regulation during metamorphosis. The developmental stages used for the experiments were as follows: before metamorphosis/prometamorphic stages (stage 56/57), at the beginning of the metamorphic climax (stage 60/61) and end of metamorphosis (stage 65/66). The tadpoles were euthanized using 0.2% aqueous tricaine methanesulfonate (MS-222), and the specimens were preserved in different fixatives or fresh tissues were taken according to each experiment. Tadpoles were staged according to Nieuwkoop and Faber (NF) [18]. The codes generated for the bioinformatics analyses are deposited in GitHub (https://github.com/GayaniSenevirathne/Senevirathne_et_al_RNAseq.git). The raw sequences are available at NCBI (GSM7701532−GSM7701554) and uploaded to Dryad (https://doi.org/10.5061/dryad.6m905qg68) [19]. Table S1 in the electronic supplementary material contains all the sample names, tissues and replicate details.

Refer to the electronic supplementary material for detailed methodology of RNA and ATAC-seq bioinformatic analyses and HCR *in situ* hybridization.

### 2.1. RNA-seq using spatial transcriptomics and laser capture microdissection

All forceps, scissors, surgical blades and lab benches were cleaned with RNAse away and 100% ethanol prior to RNA sequencing experiments. The region where the urostyle forms (demarcated by the 10th and 14th myotomes [6]) was dissected under a Leica L2 light microscope on ice-cold 1× DEPC-treated PBS; all the dissections were done on ice to prevent RNA degradation. The dissected tissue was immediately transferred to ice-cold optimal cutting temperature (OCT) compound and flash frozen in liquid nitrogen and stored at −80°C. For better RNA quality, the tissue blocks were processed the subsequent day.

**Figure 1.**
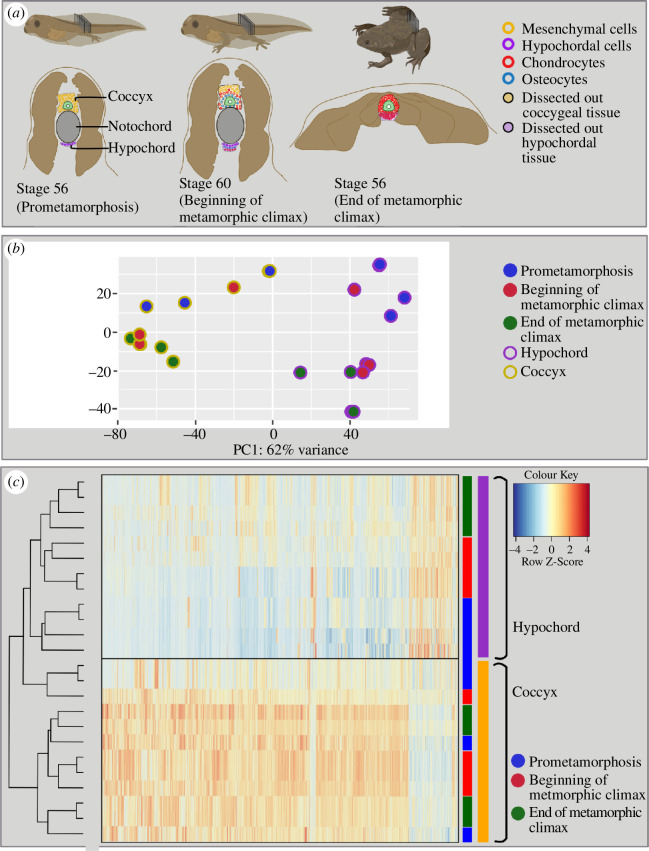
Changes in transcriptomics during anuran urostyle development**.** (*a*) The experimental setup and the developmental stages used for laser-capture microdissections. Ten sections of cryosections (10 μM each) were taken from three developmental stages (stage 56: prometamorphosis; stage 61: beginning of the metamorphic climax and stage 65: end of metamorphosis), and the coccygeal and hypochordal tissues were dissected out. (*b*) Principal component analysis for the urostyle tissues (coccyx and hypochord) used for the transcriptomic assay (*n* = 4 biological replicates per developmental stage). Each dot represents a tissue sample. (*c*) Heatmap, highlighting the differentially expressed genes, compared between three developmental stages and two tissue types. The heatmap highlights that the two tissue types possess two distinct sets of genes.

The two targeted tissue types, coccyx and hypochord, from three individuals at each developmental stage (prometamorphosis, beginning of metamorphic climax and end of metamorphosis) were dissected from frozen sections (figure 1*a*). Prometamorphic (stage 56) sections of coccyx had undifferentiated mesenchymal cells around the spinal cord, and the hypochord had embryonic hypochordal cells ventral to the notochord. The RNA-sequencing protocol followed a spatial transcriptomics method (Geo-seq; [20]). Prior to sectioning, the cryostat, brushes, adjacent benches/tabletops, blades and pencils/pens were cleaned using RNAse away and 100% ethanol. The frozen tissue blocks were sectioned using a Leica cryostat. The tissue blocks were left inside the cryostat for 20 min, allowing them to equilibrate at −20^o^C (not doing this resulted in flaky sections or sections breaking when transferred onto the slides). The tissues were sectioned at 10 μM thickness onto PEN membrane 1.0 slides. Five sections were placed on each slide and were allowed to dry at room temperature for 1 min before storing them at −80°C for further processing (the yield of RNA was high when the slides were sectioned on the same day).

On the day of the LCM, slides were thawed at room temperature for 2 min and placed under an UV lamp for 2 min to adhere sections. Next, slides were stained using Cresyl Violet to help visualize cells. For this, slides were graded through an ethanol series. Each wash was 30 s (100% ethanol, 70% ethanol, Cresyl Violet in 70% ethanol and were dehydrated in 70%, 90% and 100% ethanol). Slides were allowed to dry completely before moving to the next step (this step was important to avoid humidity affecting the RNA quality [21]).

The dehydrated slides were processed via LCM with the following settings: aperture (10), speed (20) and energy (50). The hypochordal and coccygeal cells were identified using the ×10 eye piece, and the dissections were done using the ×20. Targeted cells were captured to an adhesive cap Eppendorf tube, with the cap consisting of 50 μl of the lysis buffer. Once cells from coccyx and hypochord were collected (approx. 10 000 cells from 10 sections for each tissue type, 4 replicates were done for each stage and a total of 24 samples), 150 μl of the lysis buffer was added to each tube and the sample was left on ice for 20 min. RNA was extracted from the captured cells using the Takara NucleoSpin RNA XS (cat. no. 740902) kit with slight modification; the filtration step was skipped. cDNA was generated using the SMART-Seq v4 Ultra Low Input RNA Kit for Sequencing with the number of amplification cycles set to 18. cDNA was purified using Agencourt Ampure XP magnetic beads (Beckman Coulter) and was sequenced using the HiSeq PE100. Downstream bioinformatic RNA-seq analysis is given in detail under electronic supplementary material.

### 2.2. Gene regulation and ATAC-seq

The same developmental stages that were used for the RNA-seq studies were analysed. The urostyle region was dissected out as a fresh chunk of tissue. The OMNI-ATAC-seq protocol was used to identify open chromatin regions during urostyle development. Two replicates from each developmental stage, coinciding with the RNA-seq and morphological studies, were selected.

The tadpoles were anaesthetized in MS-222, dissected on ice-cold 1× PBS, and mechanically crushed using a pestle that had been cleaned using 100% ethanol prior to this step to obtain a homogenized sample. These steps were done on ice to prevent protein degradation. After homogenization, cells were counted using the BioRad Tc20 automated cell counter. All samples consisted of 75 000−100 000 cells. The subsequent steps followed the OMNI ATAC-seq protocol [22,23] with slight modifications using the Illumina Tagment DNA Enzyme and Buffer kit: cells were lysed in an ice-cold lysis buffer, followed by a transposition step using Tn5 Transposase, and DNA was purified using the Zymo DNA Clean and Concentrator. Purified DNA was amplified with 13 amplification cycles (the number of cycles was optimized by an additional qPCR step). Finally, the libraries were purified using the Zymo DNA Clean and Concentrator and were sequenced using a NovaSeq 2000 (100 bp paired end). The ATAC-seq bioinformatic analysis is discussed in detail under electronic supplementary material.

## 3. Results and discussion

### 3.1. Disparity in gene expression profiles of the coccyx and hypochord

**Figure 2.**
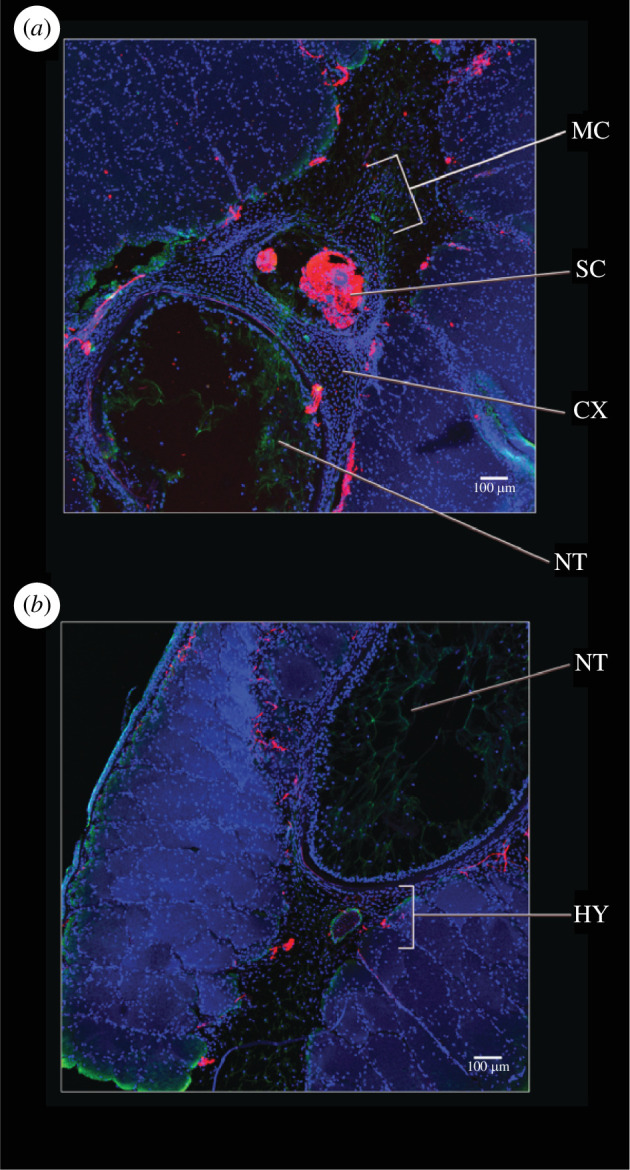
Comparison of the hypochordal and coccygeal sections before metamorphosis (stage 57)**.** (*a*) A transverse section across the coccyx, highlighting the aggregating mesenchymal cells around the spinal cord. (*b*) A transverse section across the hypochord, highlighting the embryonic hypochordal cells ventral to the notochord and notochordal sheath. Nuclei stained in blue, using DAPI and neurons stained in red using acetylated tubulin. Abbreviations: CX, coccyx; HY, hypochord; MC, mesenchymal cells; NT, notochord; SC, spinal cord.

The hypochord and coccyx show considerable differences in cellular composition and differentiation (figures 1*a* and 2), and the gene expression profiles directly reflect this (figures 1*b* *,c*). The two tissue types fuse at the end of metamorphosis, coinciding with the degeneration of the notochord. The total analysis consisted of 21 458 genes; both tissue types and the three timepoints were used as factors in the DESeq2 analysis where a binomial generalized linear model was implemented. Principal component analysis (PCA) revealed that the coccygeal and hypochordal samples generate two separate clusters (figure 1*b*), and a heatmap showed the two tissue types possess two different gene expression profiles (figure 1*c*). A total of 3298 genes were differentially expressed between the urostyle and hypochord across development (the FDR < 0.05), among these DEGs, 2828 genes were significantly upregulated and 470 were downregulated in the coccygeal region compared hypochord.

**Figure 3.**
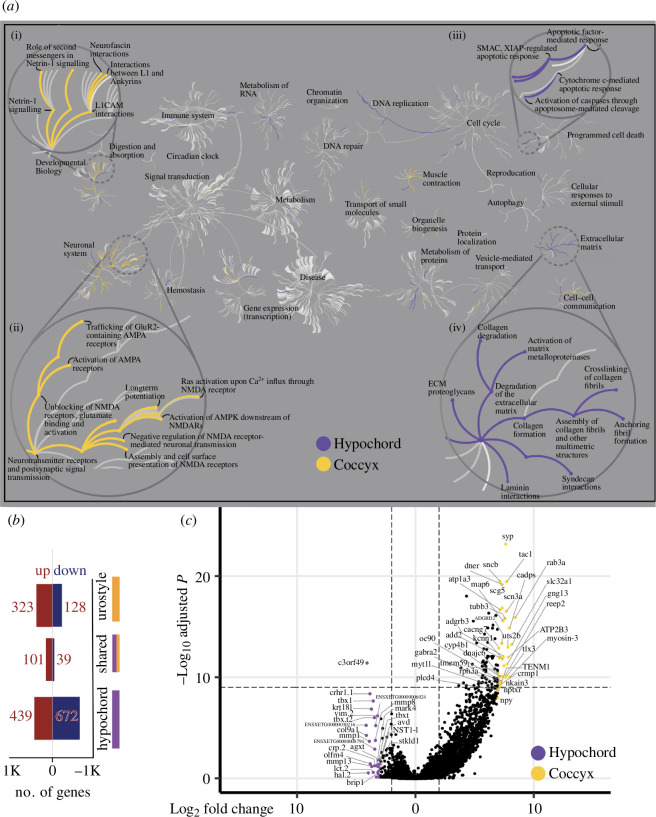
Comparative transcriptomic analysis of the two tissue types: coccyx and hypochord**.** (*a*) A reactome pathway analysis for up/down regulatory genes in coccyx versus hypochord; the central circles represent a top-level pathway, and the circles away from the centre represents lower levels in each respective pathway. ‘Zoomed-in’ sections show top-level pathways of (i) developmental biology, (ii) neuronal system, (iii) programmed cell death and (iv) extracellular matrix organization. Overrepresented pathways (*p* < 0.05) are coloured in yellow (coccyx) and purple (hypochord). Pathways that are not significant are shown in light grey lines. Most hypochordal genes are involved in organizing the extracellular matrix, whereas the majority of coccygeal genes are involved in neuronal remodelling and modifications. (*b*) The total number of urostyle-responsive genes (FDR < 0.01) is between hypochord and coccyx. (*c*) Volcano plot showing differentially expressed genes across hypochord and coccyx during development (*p* < 0.05, FDR < 0.01).

During coccyx development, the spinal cord and peripheral nerves remodel, and muscular organization changes. Consistent with these anatomical changes, we find that there are DEGs involved in differentiation and development of the nervous system (e.g. *NEUROD6*, *PRDM12*, *COCH* and *APBA2* [24,25]), genes that are expressed during skeletal muscle development (e.g. *ACTN2* [26]) and genes that are directly involved in chondrocyte/osteocyte differentiation (e.g. *RUNX2*, *COL9A1* and *SOX8*) (figure 3) [27,28].

### 3.2. Transcriptomic differences across different time points during urostyle development

There are numerous studies of metamorphic transcriptomes (e.g. [29–35]) but none on the urostyle. We first looked into urostyle-responsive transcriptomes by comparing genes that are differentially expressed in the coccyx and hypochord at different time points: Before metamorphosis versus beginning of metamorphosis (electronic supplementary material, S1B–S3) and before/beginning of metamorphosis versus end of metamorphosis (electronic supplementary material, figure S1A). This analysis identified 5664 DEGs that fell within the thresholds of FDR < 0.01 (adjusted *p*-values < 0.05 and log fold change of 1.5) and showed unique expression patterns that were significant at each time point.

Several sets of genes were upregulated and downregulated across the three developmental time points (figure 1*c*). We identified four unique clusters when the transcriptomes were compared between the three developmental time points (before and beginning of metamorphosis versus end of metamorphosis (electronic supplementary material, figure S1). Cluster A has 47 genes that were highly downregulated at the end of metamorphosis (switched off) compared with the other two time points. This cluster includes genes involved in muscle contraction and M-band stabilization in fast skeletal muscles (e.g. *TRDN* and *MYOM2I* [36,37]), skeletal development (e.g. *SOX9* [38]), response to inflammation (PTX3 [39]), filament organizing genes (e.g. KRT18.I and VIM.2 [40,41]), extracellular matrix organizing and connective tissue-strengthening (e.g. *COL9A1*, *COL8A1* and *CHAD* [42,43]) and stress regulation (*CRCH.1* [44]). The other two gene clusters, B and C (electronic supplementary material, figure S1A), comprise genes that are both downregulated and upregulated at the end of metamorphosis. Cluster C also has 15 genes that are downregulated at the end of metamorphosis, which include collagen markers (e.g. *COL9A3*) and skeletal muscle function genes (e.g. *MYL1* and *ACTN3* [45,46]). Genes that are upregulated (10 genes) are within Cluster B and are involved in mitosis (*CCNB1* [47]), development of neurons (*POU3F1* [48]) and maintenance of myelin sheath (PLP1 [49]). When before metamorphosis was compared with beginning/end of metamorphosis, clustering of the 100 top-most significant genes revealed metamorphic genes that were downregulated before metamorphosis but were upregulated during metamorphosis. Heatmap clustering revealed five main clusters (electronic supplementary material, figure S1B). Cluster A included 28 genes that were downregulated (switched off) before metamorphosis in both coccyx and hypochord, but as soon as metamorphosis was initiated, these genes were upregulated; they are involved in functions like collagen synthesis (*SERPINH1* [50]), cell cycle (*CDK6* [51]) and thyroid hormone inactivation (DIO3; [52]). Clusters B and C include genes that are switched on prior to metamorphosis and are switched off at the onset of metamorphosis: *HES8*, *FOXP2*, *EGR1*, *HOXD1*1 and *PVALB* are representative examples. Cluster D is enriched with genes that are involved in blood sugar control (e.g. *THRAP3, IGF2BP3* [53,54]), which are downregulated before metamorphosis but are upregulated at the onset of metamorphosis. This part of the transcriptomic analysis identified DEGs that are specific to the three significant time points (before metamorphosis versus onset of metamorphosis versus end of metamorphic climax).

We next explored the Gene Ontology (GO) function of these significant genes during development. The DEGs and the corresponding *p*-values from the differential expression analyses were imported into an online database of reactome pathways (‘Reactome pathway browser’) to compare the functional aspect of these genes (figure 3*a*; electronic supplementary material, figure S2). DEGs upregulated before metamorphosis were enriched for GO terms like ‘DNA replication and pre-initiation’, ‘synthesis of DNA’, ‘polymerase switching’, ‘G1/S transition’ (figure 3*a,b*), whereas the DEGs upregulated during metamorphosis include genes that function in ‘collagen formation’, ‘cross linking of collagen fibrils’, ‘*RUNX2* regulated bone development’ and ‘osteocyte differentiation’ (figures 3*a,c,d*).

### 3.3. Hypochord, metamorphosis and T-box genes

As there are no data on the genes that are expressed during hypochordal ossification, we used the DEGs identified by the coccyx versus hypochord comparisons to scrutinize this. This identified 470 genes that were uniquely upregulated only within the hypochordal tissues (they fell within the significant threshold of adjusted *p* value < 0.05 and FDR < 0.01). We saw that hypochordal tissues express high concentrations of *TBX1*, *TBXT*, *TBXT.2* and *HAND2* (figure 3*c*).

T-box genes have been implicated in early mesodermal patterning and, especially, *Brachyury*/Xbra/TBXT is essential in early mesodermal formation [55–59], and TBXT homologues across vertebrates induce the mesoderm [60,61]. T-box genes have already been identified as being pivotal components in the differentiation of the posterior axial column (e.g. [55–58,62–65]) and seem to be playing a role in hypochordal ossification as well. Xenopus has two paralogues of the gene *Brachyury*: *TBXT* (also known as *Xbra* or *T*) and *TBXT.2* (also known as Xbra3 or T2). When *Brachyury* is knocked out, it causes loss of posterior mesoderm and failure to differentiate the notochord [63,66]. However, the expression of *TBXT* and *TBXT.2* in late-developing tadpole structures has not been reported so far.

The temporal and spatial expression patterns of *TBXT* and *TBXT.2* make them good candidate genes for regulating ossification only in hypochordal tissues. We performed HCR *in situ* hybridization to examine the temporal and spatial expression patterns of *TBXT* in hypochordal ossification. *TBXT* expression is exclusively concentrated along the ossifying hypochord at the onset of metamorphosis but is not evident in prometamorphic nor at the end of metamorphic climatic tadpoles (electronic supplementary material, figure S6).

The pelvic region undergoes dramatic changes during metamorphosis, and this period is thought to represent the developmental stage that is most susceptible to predation. The underlying stress of the remodelling tissues and hormonal responses can also be seen by the increased expression of *CRCH.1* (corticosol steroid stress hormones), having a normal hormonal response to stress. Other than these genes, the hypochord also expresses significant concentrations of *VEGF* and *HAND2*. These two genes are involved in vascular development and can also be seen expressed in embryonic hypochord where VEGF plays a role in the formation of the hypochord (e.g. [13,14,67]).

### 3.4. Transcriptomic comparisons between coccyx + hypochord and other ossifying elements

Ossification happens through two major processes: endochondral (cartilaginous precursors used as a template) and intramembranous (direct ossification of the condensed mesenchymal cells) [68]. Even though coccyx and hypochord are highlighted as derivatives of two different cell populations, they both undergo endochondral ossification [6]. During this process, mesenchymal cells condense (commit to form osteoprogenitors) and aggregate to form cartilaginous precursors during early development. Cartilaginous precursors expand and cells proliferate, next the extracellular matrix is synthesized, and finally, mineralization of the matrix occurs. These steps are similar to other bones in vertebrates, which undergo endochondral ossification as well [69].

Youlten *et al*. [70] identified three clusters of GO functions during osteocyte development: (i) an 'early expression cluster’ (expressed in osteoprogenitors/osteoblast-like cells); (ii) an ‘early activation cluster’ (expressed in early osteocytes); and (iii) a 'maturation cluster’ (expressed in mature osteocytes). We compared the spatial and temporal transcriptomic maps of the osteocytes introduced by Youlten *et al*. [70] with the coccygeal and hypochordal transcriptomics to see whether the molecular underpinning of ossification is similar in the genes responsible for the formation of the urostyle as well.

*Early expression cluster*. This included GO term functions ‘extracellular matrix organization’, ‘angiogenesis’, ‘cartilage development’ and ‘connective tissue development’ (electronic supplementary material, figure S4). Out of the genes that are differentially expressed, there are some that are inactive before metamorphosis in the hypochord (e.g. *COL22A1, COL16A1, COL6A3,* RUNX1 and *IHH*) but are highly expressed once the metamorphosis is initiated. High expression of these genes in the coccygeal cells even before the onset of metamorphosis corroborates our morphological studies, where we revealed that the post caudal vertebrae of the coccyx initiated mesenchymal cell aggregation early in development (1.5 months after embryogenesis) versus 2 months in hypochord. Apart from the differences in the temporal expression of genes within the ‘early expression cluster’, a few genes involved in cartilage development are not present in the hypochord compared with the coccyx (e.g. *FOXL1*, *RUNX3*, *FOXD3*, *PMM2* and *EDN1*).

**Figure 4.**
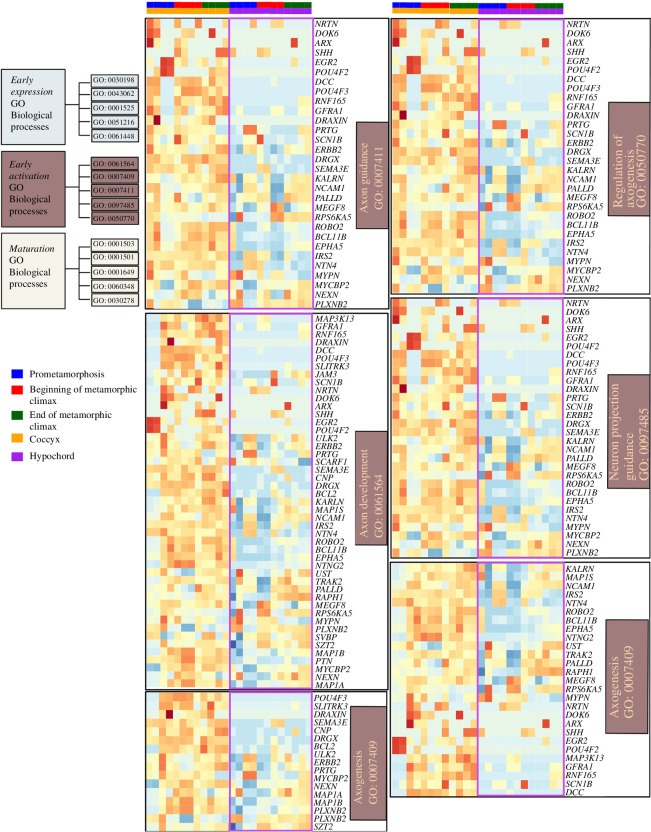
Heatmaps showing differentially expressed genes involved in GO functions belonging to the ‘early activation cluster’ of osteocyte differentiation. This cluster includes the GO functions ‘axon guidance’, ‘axon development’, ‘axogenesis’, ‘regulation of axogenesis’ and ‘neuron projection guidance’. Genes of interest that are differentially expressed between the coccyx and hypochord are highlighted in purple.

*Early activation cluster*. This cluster includes the GO terms ‘axon guidance’, ‘axon development’, ‘axogenesis’, ‘regulation of axogenesis’ and ‘neuron projection guidance’ (figure 4). While the coccyx DEGs act in a similar way to the rest of the long bones in vertebrates within this cluster, hypochord shows a different pattern. Most of the genes (e.g. *NTRN*, *SLITRK3*, *POUF42* and *DCC*) that are discussed as essential regulators in guiding the axons in long bones are not expressed within the hypochord (figure 4).

*Maturation cluster*. The GO term functions ‘bone development’, ‘skeletal system development’, ‘regulation of ossification’, ‘ossification’ and ‘osteoblast differentiation’ are included in this cluster (electronic supplementary material, figure S5). Maturation period in the hypochord happens once the metamorphosis is initiated and when the tadpole reaches the end of its metamorphic climax (electronic supplementary material, figure S5). Within the hypochord, genes involved in ossification (e.g. *GPC3*, *TMEM19*, *IFITM5*, *COL11A1*, *PHOSPHO1* and *SOX8*) and osteoblast differentiation (e.g. *GLI1, FBN2* and *SATB2*) are highly expressed in tadpoles at the end of the metamorphic climatic and are inactive at prometamorphic stages. Comparatively, in the coccyx, since the ossification happens prior to the metamorphic climax, the majority of the genes are highly expressed even at the beginning of metamorphosis. A few genes (e.g. *TBX15*, *BARX2*, *SHH* and *AXIN2*) are not expressed in hypochord nor in the coccyx, compared with the other ossifying long bones in vertebrates.

This transcriptomic comparison led to three main findings (i) Between the two tissue types, the coccyx’s DEGs share similarities with the other bones’ transcriptomics in vertebrates. (ii) Hypochord undergoes its early activation period before metamorphosis, and a maturation period once metamorphosis is initiated. (iii) Hypochordal DEGs lack an early activation period, which includes most of the axon developing genes.

### 3.5. ATAC-seq and urostyle-responsive gene regulation

**Figure 5.**
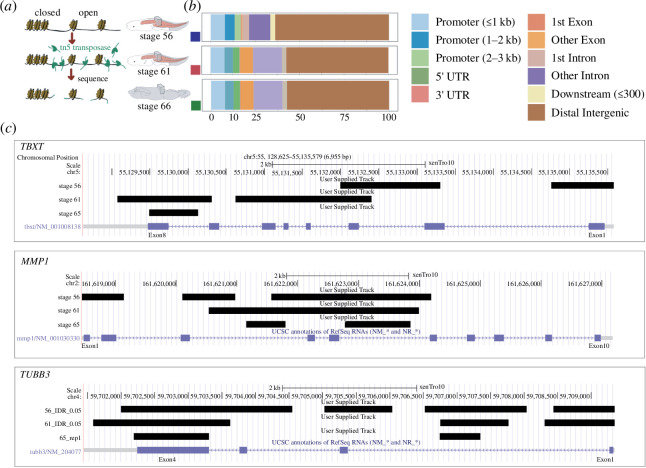
Urostyle-responsive regulatory regions. (*a*) Schematic diagram showing the workflow for chromatin profiling experiment. (*b*) Proportions of developing urostyle ATAC-seq peaks annotated to different genomic regions across development; majority of the peaks fall within the distal intergenic region and beginning (stage 61) and end of metamorphic climatic (stage 65) peaks differ from the prometamorphic (stage 56) ATAC-seq peaks with respect to peaks falling within the exon regions that are not the first exon. (*c*) Open chromatin regions (depicted using black rectangles) identified from IDR 0.05 called peaks for stage 56, stage 61 and stage 65 at the loci of validated upregulatory genes narrowed down from RNA-seq analyses.

During anuran metamorphosis, the larval body form undergoes dramatic remodelling within 6–8 days, and this is reflected in both morphological and gene expression patterns. Therefore, it can be extrapolated that gene regulation changes over this same time period. To study the underlying changes in chromatin accessibility, we used an ATAC-seq approach (figure 5*a*) using the same developmental stages and the same number of replicates as the RNA-seq work. The number of peaks varied between the three stages that we used (electronic supplementary material). More than 50% of peaks were distributed in distal intergenic regions. The rest of the peaks were distributed along intronic, exons and promoter regions. When comparing the three time points, the most significant change of peak distribution observed was the percentage of peaks that fell on the exon regions (other than the first exon): before metamorphosis, the percentage was lower (<1%) when compared with the number of peaks that were seen at the beginning and at the end of metamorphosis (7−10%) (figure 5*b*).

Next, we compared the ATAC-seq data with the RNA-seq data and observed that majority of the peaks are located close to the upregulated genes in the hypochord and coccyx that were identified from the transcriptomic data. The gene *TBXT*, which is upregulated in the hypochord, has peaks located within the exon 1, 2 and 3 before metamorphosis, and the exon 1’s peak is lost during metamorphosis (figure 5*c*). Other genes expressed in hypochordal tissues like *MMP1* also show a similar pattern where the open chromatin region changes during the course of metamorphosis (figure 5*c*). Genes that were upregulated in the coccyx, e.g. *TUBB3* shows significant peaks that are present throughout development (figure 5*c*). This could be because the coccygeal ossification occurs early in development (after 1.5 months) compared with the hypochord, which is initiated at the onset of metamorphosis. These results highlight urostyle-responsive regulatory regions during development and need further scrutinization using functional assays.

## 4. Conclusion

The coccyx and hypochord both undergo endochondral ossification during development, and similar ossification patterns were reflected in the gene expression profiles as well. Though there were major differences in some transcriptomes (e.g. presence of T-box genes, *CRCH.1* and *MMP*s in hypochordal tissues at the onset of metamorphosis versus absent in the coccyx), there were similarities in genes that were involved in endochondral ossification: we show that genes that are involved in cartilage and bone formation, extracellular matrix organization and thyroid hormone responsive elements are present in both tissues (electronic supplementary material, figure S2) but differ temporally (the coccyx starts ossifying after 1.5 months, whereas the hypochord initiates its ossification only at the onset of metamorphosis).

### 4.1. T-box genes and the hypochord

The coccyx and hypochord have two sets of differentially expressed genes. This analysis revealed a large set of genes (electronic supplementary material) that are uniquely upregulated in the hypochord and have not been reported before in the context of a developing hypochord. One of the most significant groups of genes that is upregulated in the hypochord is the T-box genes (*TBXT* and *TBXT.2*). T-box genes have a 180 bp DNA-binding domain that is highly conserved. Orthologues of the gene *Brachyury*, one of the highly expressed T-box genes in the hypochord, are present in all multicellular organisms [62]. *Brachyury* is important in posterior mesoderm development (initially expressed in the developing mesoderm but later restricted to the tail bud and notochord) [65]. While early mesoderm differentiation patterning depends highly on *TBXT*/*TBXT.2*, a role for these genes in later developmental stages has not been previously reported or discussed. We hypothesize that the presence of high levels of *TBXT*/*TBXT.2* causes the undifferentiated hypochordal cells to undergo ossification at the onset of metamorphosis. Such unusual ossification appears to also occur in response to a congenital vertebral column malformation (VCM) in humans that happens because of a *Brachyury* gene mutation in the intron 7 [64] and in the highly conserved T-box sequence [71]; these VCMs eventually lead to sacral agenesis (frog-like) syndrome in babies. In humans with this abnormality, increased expression or duplications of the *TBXT* gene result in production of excess *Brachyury* [62,72]. Apart from these mutations, *TBXT*/*TBXT.2* genes also induce EMT in humans when over expressed in carcinoma cells [73], and it has also been recorded that duplications of the *Brachyury* gene cause vertebral column chordomas [73].

Previous studies have shown that *Brachyury* acts as a switch in posterior mesoderm specification during embryogenesis and is restricted to the anteroposterior axis [55,56]. Here, during hypochordal ossification, the onset of metamorphosis could be triggering ectopic expression of *TBXT/TBXT.2* in hypochordal cells, which could potentially express posterior mesodermal genes and subsequently activate downstream targets of *TBXT/TBXT2*, which in turn initiates chondrification and ossification. When *Brachyury* genes are highly expressed in human chordoma cells, matrix metalloproteinases (e.g. *MMP12, MMP13* and *MMP24*; 74) are also upregulated at the same time (which is also seen in hypochordal cells). The extent to which the human and frog conditions are similar awaits functional tests.

### 4.2. Coccyx and hypochord versus other vertebrate skeletal elements

The coccyx and hypochord undergo endochondral ossification and show an array of genes that are similar to the genes expressed in other long bones that undergo endochondral ossification in vertebrates (e.g. mesenchymal-to-chondrocytes involved genes like *BMPs, SOX9*; chondrocytes-to-osteoblasts/osteocytes were seen in highly expressed genes like *RUNX2*, Osterix and *IHH*). Apart from these similarities, when comparing the already published osteocyte transcriptomics [70], hypochord shows some considerable differences among the rest of the bones in vertebrates. Hypochordal cells express osteoprogenitor-specific genes before the metamorphic climax, and metamorphosis acts as a switch that activates osteogenesis (versus in coccyx osteogenesis is initiated prior to metamorphosis). Other than the temporal differences observed regarding ossification, the DEGs of the hypochord reveal that hypochordal cells lack the ‘early activation phase’, which includes regulators needed in ‘Axogenesis’ and ‘Axon development’ in ossifying bones (figure 4). Vertebrate bones are innervated by sensory and sympathetic nerves during skeleton development [75], where the periosteum and bone marrow have the highest density of nerves whereas the mineralized matrix has very few [75–77]. During development, bone innervation and endochondral ossification happen simultaneously [75], and it is hypothesized that axon guidance regulates formation of the neuronal network, which is subsequently required for the osteocyte network formation [70]. It is surprising that the ossifying hypochord lacks the genes needed for axon development (figure 4), and our results raise the possibility that the hypochordal development may be disconnected from the neuronal signals. Future work is needed scrutinizing the innervation patterns within the hypochord during its development to better understand this.

Our integrative approach, using morphological and molecular datasets (genes and gene regulation) on the development of the urostyle, scrutinizes the evolution of a novelty. Future work targeting the candidate genes responsible for the development of the urostyle, together with functional assays, will shed light on the evolution of this structural enigma.

## Data Availability

The raw sequences are available at NCBI (GSM7701532-GSM7701554) and uploaded to Dryad [78]. Table S1 of the electronic supplementary material contains all the sample names, tissues and replicate details [79].
